# Design and MinION testing of a nanopore targeted gene sequencing panel for chronic lymphocytic leukemia

**DOI:** 10.1038/s41598-018-30330-y

**Published:** 2018-08-07

**Authors:** Paola Orsini, Crescenzio F. Minervini, Cosimo Cumbo, Luisa Anelli, Antonella Zagaria, Angela Minervini, Nicoletta Coccaro, Giuseppina Tota, Paola Casieri, Luciana Impera, Elisa Parciante, Claudia Brunetti, Annamaria Giordano, Giorgina Specchia, Francesco Albano

**Affiliations:** Department of Emergency and Organ Transplantation (D.E.T.O.), Hematology Section, University of Bari, 70124 Bari, Italy

## Abstract

We report a customized gene panel assay based on multiplex long-PCR followed by third generation sequencing on nanopore technology (MinION), designed to analyze five frequently mutated genes in chronic lymphocytic leukemia (CLL): TP53, NOTCH1, BIRC3, SF3B1 and MYD88. For this purpose, 12 patients were selected according to specific cytogenetic and molecular features significantly associated with their mutational status. In addition, simultaneous analysis of the targets genes was performed by molecular assays or Sanger Sequencing. Data analysis included mapping to the GRCh37 human reference genome, variant calling and annotation, and average sequencing depth/error rate analysis. The sequencing depth resulted on average higher for smaller amplicons, and the final breadth of coverage of the panel was 94.1%. The error rate was about 6% and 2% for insertions/deletions and single nucleotide variants, respectively. Our gene panel allows analysis of the prognostically relevant genes in CLL, with two PCRs per patient. This strategy offers an easy and affordable workflow, although further advances are required to improve the accuracy of the technology and its use in the clinical field. Nevertheless, the rapid and constant development of nanopore technology, in terms of chemistry advances, more accurate basecallers and analysis software, offers promise for a wide use of MinION in the future.

## Introduction

Chronic lymphocytic leukemia (CLL) is a disease with a highly heterogeneous clinical course, characterized by the accumulation and proliferation of clonal B cells in the blood, bone marrow and lymph nodes. Next–generation sequencing (NGS) technologies applied to genome-wide association studies (GWAS) have revealed several recurrent gene mutations associated with CLL that, in association with the high level of genetic heterogeneity among patients, is consistent with the large degree of clinical variability characteristic of this disease. In fact, a number of recurrent gene mutations have been reported in 2–10% of newly diagnosed CLL cases^1^, and clinical evidence revealed the prognostic role of specific mutated genes in defining different molecular subtypes of CLL. They may help to predict the potential evolution of the disease or the response to therapy. Identifying the most clinically and biologically relevant clones is crucial for evaluating CLL disease progression; in this context, NGS targeted gene sequencing allows driver mutations to be detected, at diagnosis and/or responsible for progression and relapse^2,3^, which could be used as predictive or prognostic biomarkers in CLL^4^.

However, the introduction of NGS technologies in clinical diagnostics requires a high initial investment when purchasing the sequencer, that is a limitation for local research facilities in developing countries and small research centers and hospitals. A valid alternative could be the use of MinION, the first commercially available sequencer based on nanopore technology. MinION has already been successfully used to detect mutations of the *TP53* and *ABL1* genes in CLL and in chronic myeloid leukemia (CML) patients, respectively^5,6^. Moreover, the very low costs, the ease of use, and the length of the reads make MinION an ideal tool for targeted sequencing of genes^5–7^.

In this report we describe the development of a customized gene panel, MinION-based, for targeted sequencing of the five genes TP53, BIRC3, NOTCH1, SF3B1 and MYD88, recurrently mutated in CLL, and analysis of its performance.

## Results

### Real-time data monitoring and error rate analysis

In our experiment, the library consisting of 12 barcoded samples was loaded for sequencing on MinION, employing a flowcell with 1,170 active pores. A total of 106,543 fast5 total files was produced, containing raw electric signals, for a total of about 65 Mb. Fast5 files were then uploaded in Metrichor for basecalling and demultiplexing during the run. Of the total reads produced, 48,599 passed 2D filters, and 46,451 had a recognizable barcode. Only “pass” reads were used in the study; the number of 2D passed reads for each sample is illustrated in Supplementary Table S1.

Thanks to the real-time analysis of the fast5 files produced, after 24 hours from the start of the sequencing we observed that the minimum sequencing depth of the targets for case #7 was lower, on average, than for the other CLL samples. Therefore, we decided to prepare and reload a new pool comprising all the libraries at different weight ratios according to the preliminary results obtained with real-time sequencing monitoring. Employing this expedient, the total pass 2D reads count was greater than 2,000 for each sample except for case #7 (Table 1). Plotting the distribution of read lengths, all passed 2D reads of each sample had a length corresponding to the expected amplicons size (Supplementary Fig. S2).

**Table 1:** CLL patients clinical data and reads mapping analysis (del(11q), 11q deletion; del(13q). 13q deletion; del(17p), 17p deletion).

Sample	Sex/Age	FISH	IgVH status	Sample type	2D mapped reads count/ total 2D reads count
CLL#1	F/63	88% del(13q), 96,5% del(11q)	Unmutated	Peripheral blood	3013/3066 (98.27%)
CLL#2	M/73	83% del(13q), 8% del(17p), 66% del(11q)	Unmutated	Peripheral blood	4752/4859 (97.80%)
CLL#3	F/62	42% del(11q)	Unmutated	Peripheral blood	2254/2272 (99.21%)
CLL#4	M/72	50% del(11q), 80% +12q13	Unmutated	Peripheral blood	3207/3234 (99.17%)
CLL#5	M/50	71% del(13q), 97,1% del(11q)	Unmutated	Bone marrow	3845/3919 (98.11%)
CLL#6	F/53	51% +12q13	Unmutated	Peripheral blood	4432/4593 (96.49%)
CLL#7	M/74	65% +12q13	Mutated	Peripheral blood	1450/1460 (99.32%)
CLL#8	F/71	89% del(13q), 91% del(17p)	Unmutated	Peripheral blood	5392/5483 (98.34%)
CLL#9	M/79	87% del(13q) (biallelic), 84% del(17p)	Mutated	Peripheral blood	4959/5106 (97.12%)
CLL#10	M/58	74% del(13q)	Mutated	Peripheral blood	3428/3653 (93.84%)
CLL#11	M/59	62% +12q13	Unmutated	Peripheral blood	3232/3345 (96.62%)
CLL#12	M/62	negative for del(13q), del(17p), del(11q), +12q13	Unmutated	Peripheral blood	2480/2535 (97.83%)

NanoOK analysis was performed to calculate the error rate for each amplicon. In Table 2, the mean values of identical, inserted and deleted bases, and substitutions per 100 aligned bases (including indels) are shown for each amplicon. As observed, separate error rate analysis for insertions, deletions and substitutions revealed higher error values for deletions, in line with historically known nanopore error rate data^8–10^.

**Table 2:** Error rate analysis of MinION sequencing data. As observed, for all the targets of the CLL panel the mean error rate calculated was higher for deletions than for insertions and substitutions (**Indels**: insertions/deletions).

Targets	Mean identity per 100 aligned bases (including indels)	Mean insertion error rate	Mean deletion error rate	Mean substitution error rate
BIRC3 exons 7–9	92.86%	0.91%	4.47%	1.76%
TP53 exons 10–11	90.83%	0.90%	6.26%	2.02%
NOTCH1 exon 34–3′ UTR	90.50%	0.86%	6.58%	2.09%
SF3B1 exons 14–16	92.75%	0.89%	4.56%	1.80%
TP53 exons 2–9	90.09%	0.80%	7.19%	1.93%
MYD88 exons 3–5	91.09%	0.89%	5.98%	2.04%
BIRC3 exon 6	93.45%	0.74%	4.56%	1.25%
Mean	**91.65%**	**0.86%**	**5.66%**	**1.84%**

### Sequencing depth analysis

**Figure 1:**
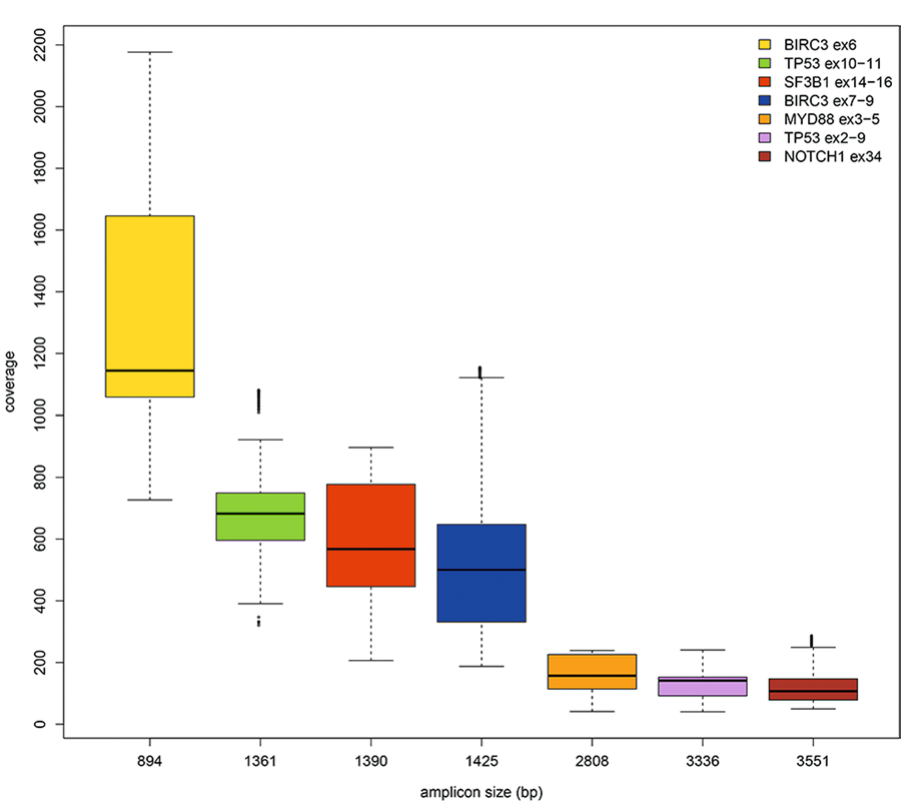
Boxplot of sequencing depth data and amplicons size (bp). The range of read depth was more uniform for longer amplicons and inversely related to the amplicon size, the smaller amplicons showing a higher sequencing depth.

Analysis of NanoOK data showed that the range of sequencing depth was more uniform for longer amplicons, and, notably, inversely related to the amplicon size, the smaller amplicons having a greater depth (up to about 2100x) (Fig. 1). These data suggest that, in the case of amplicons of variable size and smaller than 3 kb, further adjustments should be made in the adapter ligation step of MinION library preparation, in order to ensure a more uniform depth rate. In any case, except for case #7, the minimum sequencing depth value was never below 50x for all the amplicons analyzed; therefore, we decided to exclude this sample from the subsequent analyses.

### Performance evaluation of MinION sequencing for CLL hotspot mutations

We also focused on the known hotspot mutations in CLL, NOTCH1 p.P2514fs*4^11,12^, SF3B1 p.K700E, p.G742D, p.K666 and p.H662^12,13^, and MYD88 p.L265P^14^, and calculated the average depth of coverage in these specific genomic positions. For all hotspot mutations, the minimum sequencing depth value was above 50×, and reflected the range of read depth related to amplicons sizes. Examining the mean and substitutions/deletions/insertions error rates in these positions, we observed a comparable trend to the general error rate analysis, with higher error rates for deletions than for substitutions or insertions. Supplementary Table S2 reports the error rate/depth of coverage data of the known hotspots.

### Variant calling, filtering and annotation

We performed two parallel analyses of MinION sequencing data, uploading the fastq files extracted from fast5 files to Galaxy platform or analyzing them with the Nanopolish variant calling pipeline^15^, by applying similar settings (see Material and Methods). In detail, we created and used a complete workflow (https://usegalaxy.org/u/ematlab/w/mutation-detection-llc-panel), including read mapping, variant calling and annotation. Similarly, the Nanopolish pipeline was applied starting from fast5 files to variant calling, and then the ANNOVAR annotation tool^16^ from Galaxy was used to annotate them. For both the workflows, SNV and indels analysis was performed for each CLL case.

SNV and indels were filtered for hotspot mutations or genomic location (exonic and splicing variants), functional effect (nonsynonymous variants), and frequency based on minor allele frequency (maf) from the 1000 Genomes Project (<1%). Variant allele frequency cut-off was set at 10% and 15% for SNV and indels, respectively, in agreement with the recent results of serial dilution for MinION somatic mutation analysis^17^. The filtered variants were also analysed *in silico* by the pathogenicity prediction program SIFT incorporated in ANNOVAR.

**Figure 2:**
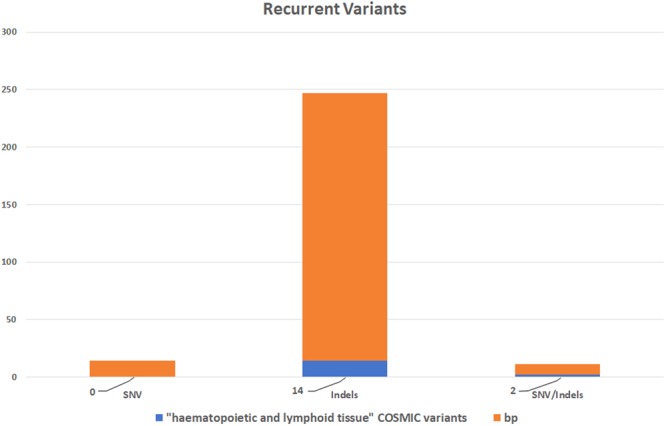
Plot of the recurrent variants identified in the CLL patients analyzed and filtered out from the analysis. Most of them are sites of indels (nucleotides count orange). A small fraction of these critical positions (about 7%) are annotated in COSMIC v81 as identified in haematopoietic and lymphoid tissue (blue).

It is well-known that nanopore sequencing reads still have a high error rate, especially in homopolymer contexts^9^. To reduce the risk of producing false positive results due to sequencing errors, we checked if the same variants were called in different patients. To this aim, we calculated the recurrence of all SNV and indels and found some recurrent variants in several samples, and even in all 12 CLL cases. Except for the known CLL mutation hotspots, and the polymorphisms identified, considering the low likelihood of finding the same variant simultaneously in a small cohort, we decided to exclude the variants occurring in multiple samples from further validation analyses. In total 256 genomic recurrently mutated positions were therefore determined; most of them (91%) were sites of indels, in line with the results of the error rate analysis (see Material and Methods), whereas 9 were sites of both SNV/indels and 14 only of SNV (Fig. 2, Supplementary Table S3).

Excluding these critical positions from the analysis, the actual breadth of coverage of the custom panel dropped from 100% of the long amplicons covering all the target regions to 94.7%. Indeed, 5.3% of the genomic positions covered with the amplicons design were sites of recurrent errors, determining a high fraction of false positive results in these positions. These data are not related to panel design, but rather to the chemistry and base calling algorithms used (See Material and Methods).

We also verified whether these critical positions are known to be sites of experimentally observed mutations in haematopoetic and lymphoid tissue and found that about 7% of them were COSMIC (v81) variants annotated in haematological malignancies (Fig. 2). These filtered-out COSMIC variants did not include the known hotspots listed above.

We also retrieved the nucleotide sequences flanking of all the SNV/indels called and observed that most of these mutations suggested a context-specific error frequently caused by homopolymer sequences (Supplementary Table S3).

**Figure 3:**
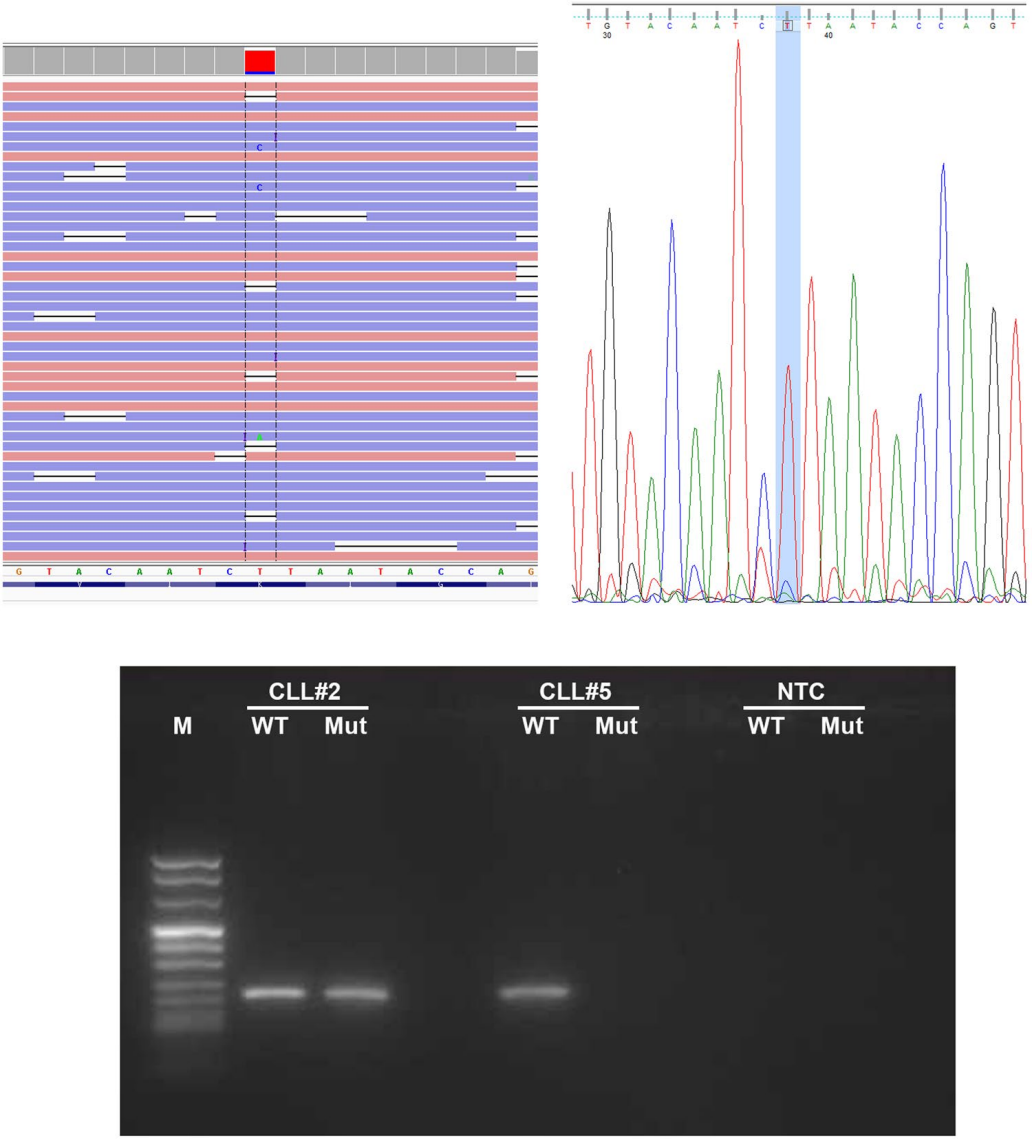
The SF3B1 mutation identified in case#2 thanks to MinION and validated by ASO-PCR. Aligned reads are visualized by the Integrative Genomics Viewer (IGV) browser (on the left). The genomic position, and the depth of sequencing for each base are reported as a gray bar. Variants with an allelic ratio >10% are reported as colored depth of sequencing bars, where each color represents the specific base fraction. As shown, the mutation was barely visible in SS (on the right) and was validated by ASO-PCR (at the bottom). The original image of the gel is provided as Supplementary Fig. S4.

For these reasons, we decided to focus on the non-recurrent variants and known hotspots: 18 SNV and 6 indels (Supplementary Table S4). We compared these mutations revealed by MinION with the results of SS and molecular assays: 8 pathogenic mutations were finally confirmed by SS or other molecular assays in 6 patients, with 2 patients concurrently harboring 2 mutations. In detail, 6 mutations were SNV, whereas the main hotspot deletion of NOTCH1 p.P2514fs*4 was detected in 2 patients (Table 3). These mutations were simultaneously identified with SS except for 2 variants having a mutation allelic ratio below the detection limit of SS: for case #4, one mutation in the coding region of BIRC3, previously not easily detected by SS electropherogram analysis, was detected by visual inspection on the indications generated by MinION, whereas in case #2 a mutation in SF3B1 was validated by ASO-PCR (Fig. 3, Supplementary Fig. S3).

**Table 3:** Genomic description and annotation of mutations detected by MinION sequencing or other molecular assays (**ASO-PCR**: Allele Specific Oligonucleotide PCR; **SS**: Sanger Sequencing).

Patient	Gene	Variant	Hotspot mutation	Mutation type	Protein description	Impact	Allelic Ratio (%)	Validation method	Varscan detection	Nanopolish detection	Depth of coverage
CLL#2	SF3B1	chr2:198267360T>C	yes	SNV	p.K666R	MODERATE	11.75	ASO-PCR	yes	no	722
CLL#2	NOTCH1	chr9:139390721G>C	yes	SNV	p.Y2490X	HIGH	27.14	SS	yes	no	73
CLL#4	BIRC3	chr11:102206703T>G	no	SNV	p.L444X	HIGH	10.04	SS	yes	no	323
CLL#5	TP53	chr17:7577022G>A	no	SNV	p.R306*	HIGH	84.14	SS	yes	yes	156
CLL#8	TP53	chr17:7577121G>T	yes	SNV	p.R273G	MODERATE	47.54	SS	yes	yes	70
CLL#8	NOTCH1	chr9:139390649AG/—	no	INDEL	p.P2514fs*4	HIGH	20.69	SS	yes	no	62
CLL#9	TP53	chr17:7578478G>C	no	SNV	p.P19R	MODERATE	57.50	SS	yes	yes	91
CLL#12	NOTCH1	chr9:139390649AG/—	yes	INDEL	p.P2514fs*4	HIGH	20.69	SS	yes	no	86

Applying the Nanopolish pipeline, we confirmed only 3 of the 8 variants previously called with Varscan, suggesting that Nanopolish is more stringent for variants detection, less sensitive than Varscan, and can give false negatives (Table 3). The lower sensitivity of Nanopolish for detecting variants was due to the computation of an improved consensus sequence before variant calling rather than to the more stringent parameters used. Indeed, this step is part of the Nanopolish pipeline, and probably makes the identification of low allele-frequency variants more difficult. In accordance with this, all the variants called in our samples with Nanopolish showed high allele frequencies (Table 3).

## Discussion

MinION is a low-cost, handheld sequencer that produces long reads (recently up to megabases) in real-time^18,19^. Nowadays, MinION can generate up to around 5–10Gbp of DNA sequence data, and has been used in particular to sequence and assemble microbial, yeast and fungi genomes^20,21^. Recently, MinION has also been used to sequence Ebola, chikungunya, and hepatitis C viruses using an unbiased metagenomic approach with randomly amplified cDNA^22^. Analysis of genomic mutations in cancer^5,6,17,23,24^ and in the infectious diseases field^25^ has been reported using MinION. In these studies nanopore technology was employed to detect mutations in a single gene target, or in several targets after PCR products pooling, applying slightly different methods^17,25^.

To the best of our knowledge, this is the first report of targeted gene sequencing based on a custom panel of pre-pooled primers for multiplex long-PCR on MinION.

Multiplex gene panels are one of the strategies most commonly used to enrich the genomic regions to be sequenced, and are widely employed in NGS technologies. This approach, applied to MinION, allowed us to enrich all the gene regions of interest, setting up only 2 PCR for each sample and thus simplifying the experimental procedures.

Thanks to MinION real-time sequencing, reads can be analysed as they are generated, significantly speeding up the analysis of results and modifying the experimental conditions if necessary; this advantage allowed us to intervene in real-time on the quality of case #7 sequencing. Other advantages of MinION include its portability and easy setting for library preparation and sequencing as compared to second-generation sequencers, as well as its cost-effectiveness.

Thanks to analyses of the utilization and impact of NGS in CLL, in the last years several custom/academic or commercial CLL target panels have been developed, most of them based on the amplicon sequencing approach. For example, in 2013 Kolhman *et al*. described their 13-gene panel for investigating CLL NGS, proposing it in routine diagnostics; the authors declared that using their panel the detection of the main NOTCH1 hotspot p.P2514fs*4 was successful, but that it was not able to identify larger indels^26^. Currently, only one commercial amplicon-based CLL gene panel is available which allows the detection of both SNV and copy number variant analysis for the nine most prognostically relevant genes in CLL^2^. Nonetheless, attempts to design a comprehensive gene panel useful for CLL prognostication and therapy-effect prediction, are still continuing^2^.

All these approaches have surely proven more accurate than nanopore sequencing, allowing even low allele frequency mutations (subclonal) to be detected, because of their lower error rate. Furthermore, higher throughput allows the analysis of more samples and/or more genes per sample. However, some considerations about short-read sequencing platforms should be made. Firstly, although their accuracy is very high (>99.5%), they are still affected by specific sequencing errors: Illumina displays a tendency towards substitution errors, whereas Ion Torrent technology fails when measuring homopolymers^27^. Secondly, some weaknesses related to the general short-read sequencing approach are well known: the technical limit of detecting larger indels and other structural variations (SV), and the impossibility of conducting the phasing analysis of mutations. In fact, SV larger than the read length cannot be detected and, using genomic DNA, the mutations may be too distant to be detected on the same read.

By exploiting the long reads produced by MinION sequencing, it is possible to evaluate the phasing of mutations, clarifying the allelic context of mutations affecting the same gene, but too geographically distant to be detected with short reads sequencing; this analysis may be particularly useful for CLL patients in the context of disease progression and therapy resistance. For example, in the case of no co-occurrence between mutated *TP53* and del(17p)^12^, phasing analysis of multiple mutations would allow the physician to establish whether both TP53 alleles are mutated or not^28^.

In perspective, we could also consider implementing our panel to identify even the structural alterations frequently identified in CLL patients, obtaining the complete CLL mutational spectrum in a single MinION experiment.

All genes included in our panel have a prognostic significance in CLL. Mutations of TP53 are found in 4–37% of CLL patients, generally occur within the DNA-binding motifs (exons 4–8), and have been associated with very poor prognosis in several studies^29,30^. In a recent report our group demonstrated that nanopore technology shows a correlation with SS results but is more sensitive, and has therefore proven to be a useful tool for TP53 gene mutation detection^5^. NOTCH1 gene mutations in CLL mainly cluster within the p.P2514fs*4 hotspot, and have independently conferred adverse overall survival (OS) in multiple studies, whereas their impact on progression-free survival has been inconsistent^31–33^. Moreover, NOTCH1 mutations have also been identified as a predictive marker for patients who are less likely to respond to the addition of rituximab to therapeutic regimens^34^. Mutations in the SF3B1 gene cluster in the selected HEAT repeats of the SF3B1 protein, and have been identified in 4–12% of CLL patients, with a higher frequency at the time of disease progression^13^; SF3B1 mutations are correlated with a shorter duration of remission after treatment, fludarabine-refractory disease, and poorer OS^35,36^. BIRC3 and MYD88 gene mutations have been detected in up to 4% of CLL cases^14,37^; while gene mutations in the former are associated with other adverse prognostic factors that predict chemo-refractoriness and poorer prognosis^37^, the MYD88 gene mutations, mostly L265P, identify CLL patients with a more favorable outcome^14^.

Our proof-of-concept demonstration of targeted gene sequencing in CLL using the MinION sequencer reveals the feasibility of this approach but identifies specific challenges to be dealt with in future projects. Indeed, in the last 2 years five different MinION chemistry versions (R6.0, R7.0, R7.3, R9 and R9.4), and several bioinformatics tools have been presented, improving MinION’s performance, but, despite these efforts and progress, the main weak point of MinION, namely its high error rate especially in homopolymeric sequences, still persists. In this context, basecalling is a crucial step, because it can hugely affect MinION sequencing data. To date, several basecallers have been developed to improve read accuracy^38^, and new bioinformatic pipelines are being tested, also for variant calling analysis^23,39^.

Regarding the MinION error rate, we approached this issue by excluding from the analysis those genomic positions recurrently detected as mutated in the samples analyzed, admitting the inability to evaluate the mutational status of these genomic positions with the data analysis tools currently available. By retrieving the adjacent bases of these genomic positions, most of the false positive were within or next to homopolymer sequences. This strategy clearly reduced the total actual breadth of coverage, but produced more reliable results, and did not interfere with the detection of the CLL mutation hotspots. The main NOTCH1 hotspot p.P2514fs*4 is a particular case because it involves an “AG” dinucleotide deletion followed by a “G” homopolymer which does not seem to affect its correct identification.

Today, other single-molecule platforms are also being developed in order to overcome the limit of the nanopore error rate and detect very low-frequencies point mutations^40,41^. In the future, the implementation of these innovative approaches in MinION sequencing may contribute to better discriminate between mutated and wild-type DNA molecules and will be extended to achieve the concurrent detection of multiple biomarker mutations.

In conclusion, although the sample number in our study is relatively small, and an extension of the case series is warranted to correctly estimate the sensitivity and specificity of our assay, our data showed that the CLL gene panel provides a satisfactory performance. We propose a workflow that can potentially enable laboratories equipped with only basic molecular biology techniques to perform detailed targeted gene sequencing analysis in CLL patients. In the next future, we plan to evaluate how we can scale up the target panel, including more amplicons in a larger patients sample size using MinION. The constant improvement of nanopore technology will likely result inits widespread application in clinical practice.

## Material and Methods

### Patients

The study included 12 newly diagnosed CLL patients. Patients were selected according to the presence of the genetic aberrations 11q, 13q and 17p deletions (del(11q), del(13q), del(17p)), and trisomy 12, frequently associated with CLL and identified by Fluorescent *In Situ* Hybridization (FISH), as previously reported^42–44^, and the immunoglobulin heavy-chain variable (IgHV) mutational status. These clinical parameters are significantly associated with the mutational status of the genes included in our panel^11,35^. Only one case, CLL#12, showed none of the frequent genetic aberrations analyzed in FISH analysis. All samples included in the study were characterized for the NOTCH1 hotspot p.P2514fs*4 and MYD88 hotspot p.L265P by molecular assays. For the exons of BIRC3, TP53 and SF3B1 included in the panel, mutational status was verified by Sanger Sequencing (SS). These evaluations were performed in concomitance with the MinION sequencing analysis. All 12 samples were analyzed by MinION and SS or molecular assays in a blinded manner.

Genomic DNA was extracted from peripheral blood using the QIAamp DNA Blood Mini Kit (Qiagen) and the DNA concentration and purity were checked using the Qubit 2.0 fluorometer (Life Technologies) and Nanodrop UV-Vis spectrometer (Thermo Fisher Scientific).

Patients clinical and biological characteristics are summarized in Table 1, which also illustrates the ratio of the 2D mapped reads count to the total 2D reads count for each sample.

The study complies with the current ethical guidelines, and has been approved by the Azienda Ospedaliero-Universitaria Consorziale Policlinico of Bari (Bari, Italy). The written informed consent was obtained from the patients included in this study, and patient records/information was anonymized and de-identified prior to analysis.

### CLL panel preparation and testing

Our customized CLL panel included the known mutation hotspots of SF3B1^12,13^, NOTCH1^12,32^ and MYD88^14^, and the genomic regions of the five genes reported in Table 4. The enrichment strategy we adopted to primarily select our targets was multiplex long-PCR. Amplicons were designed to cover one or multiple exons of the five genes TP53, BIRC3, NOTCH1, SF3B1 and MYD88; primer design was performed using Primer3 software (http://primer3.ut.ee/) with the following non-default parameters (*T*_min_ 59 °C, *T*_opt_ 60 °C, *T*_max_ 61 °C), to yield product sizes greater than 800 bp. The FastPCR (http://primerdigital.com/fastpcr.html) and Multiple Primer Analyzer (Thermo Fisher Scientific) tools were used to analyze and compare multiple primer sequences simultaneously. Overall, seven pairs of primers were selected for the selected target genes and analyzed with the Multiplex 2.1 tool (http://bioinfo.ut.ee/multiplx/) using the default parameters, to evaluate primers compatibility and find the best primers pooling solution. Two primers pools were thus identified: pool 1 included primers for BIRC3 exons 7–9, TP53 exons 10–11, SF3B1 exons 14–16 and NOTCH1 exon 34 including 3′UTR, while pool 2 was assembled with primers for TP53 exons 2–9, BIRC3 exon 6 and MYD88 exons 3–5. The total panel size was about 15 kb. In Table 4 the composition of the two primer pools and the respective amplicon sizes are shown; primers sequences are shown in Supplementary Table S5.

**Table 4:** Composition of the 2 pools of the custom CLL gene panel, with the relative size of the corresponding amplicons.

	Gene target	Amplicon size (bp)
Pool1	BIRC3 exons 7–9	1425
	TP53 exons 10–11	1361
	NOTCH1 exon 34–3′ UTR	3551
	SF3B1 exons 14–16	1390
Pool2	TP53 exons 2–9	3336
	MYD88 exons 3–5	2808
	BIRC3 exon 6	894

### Multiplex long-PCR target enrichment

For each CLL sample two multiplex long-PCRs were performed with the two primer pools using PrimeSTAR GXL DNA Polymerase (Takara Bio Inc.), 70 ng of genomic DNA, in a final volume of 25 uL. Thermal cycling conditions were 98 °C for 10 minutes, 60 °C for 15 seconds, 68 °C for 4 minutes (35 cycles) and 12 °C hold for both the two long-PCR.

Since 3 amplicons of pool 1 had a very similar size of about 1.3 kb and were not easily distinguishable by 1% agarose gel electrophoresis, a restriction enzyme of these critical amplicons was made, and the BglII restriction enzyme (10,000 units/mL, New England BioLabs Inc.) was finally selected to verify their successful amplification and discriminate them. In detail, 5 uL of the primer pool 1 PCR products were incubated with 0.5 units of BglII and 1uL of NEBuffer 3.1 in 1 hour at 37 °C. Digestion products were visualized by SYBR Safe on agarose gel 1%.

PCR products from both the long PCR were purified with the QIAquick PCR Purification Kit (Qiagen) in an elution volume of 30 uL, and the DNA concentration and purity was measured with a Qubit 2.0 fluorometer (Life Technologies) and Nanodrop UV-Vis spectrometer (Thermo Fisher Scientific).

Two uL of pool 1 and pool 2 purified amplicons were visualized by SYBR Safe on agarose gel 1.0% (Supplementary Fig. S1).

### Library preparation and sequencing

For each sample, 300 ng of PCR purified products from each long-PCR were pooled, in a final volume of 25 uL in nuclease-free water and used for library preparation. According to the 2D Native barcoding genomic DNA (SQK-LSK 208) protocol, the amplicons were end-prepared using the NEBNext Ultra II End Repair/dA-Tailing Module (New England Biolabs Inc.) and barcoded with the ligation of nanopore-specific Native Barcodes (NB01-NB12) using Blunt/TA Ligase Master Mix (New England Biolabs Inc.). Equimolar amounts of each barcoded amplicon were then pulled and 540 ng of the final pool were diluted to 58 μl in nuclease-free water and prepared for sequencing with the ligation of Native Barcoding adapters and the tether using NEBNext Quick Ligation Module (New England Biolabs Inc.). All purifications were performed with Agencourt AMPure XP beads (Beckman Coulter Inc.). Dynabeads MyOne Streptavidin C1 (Thermo Fisher Scientific) were used to elute the library in the pre-sequencing mix.

After the Platform QC run and the priming of the flowcell, the sequencing mix (37.5 μl of Running Buffer with Fuel Mix, 25.5 μl of Library Loading Bead Kit, 12 μl of the Pre-sequencing Mix) was loaded and the MAP_48Hr_Sequencing_Run.py protocol was started (MinION flowcell: FLO-MAP106).

Real-time analysis of the sequencing files generated by MinION was performed using a custom script including fast5 extraction, mapping with the BWA-MEM aligner and counting of the mapped reads; for each amplicon, the minimum number of reads per amplicon considered for the real-time analysis was 100x.

The sequence data from this study have been submitted to the NCBI Short Read Archive (https://www.ncbi.nlm.nih.gov/sra) under accession n° SRP133465.

### Data Analysis

During MinION sequencing, DNA bases were called using a cloud-based software (Metrichor), generating FAST5 files, from which FASTQ files were extracted with the Poretools toolkit 0.6.0.

The NanoOK tool (v.1.14) was employed to analyze the read depth and error rate, using the FASTA sequences of the target amplicons as reference. Depth of coverage and error rates of the known hotspots were calculated with bam-readcount program and by visual inspection of bam files with the Integrative Genomics Viewer (IGV) browser^45,46^.

Data analysis from reads mapping to variant calling was performed in parallel using Galaxy, a web-based platform for processing NGS data (https://usegalaxy.org/), and Nanopolish, a package specific for signal-level analysis of nanopore sequencing data (https://github.com/jts/nanopolish).

In Galaxy, reads were aligned on GRCh37 human reference genome with the BWA-MEM method using specific Nanopore platform parameters (https://github.com/lh3/bwa/blob/master/NEWS.md/#release-079-19-may-2014); the leftalign utility from FreeBayes package was applied to homogenize the positional distribution of insertions and deletions. The BAM files obtained were visualized. Single nucleotide variants (SNV) and insertions/deletions (indels) detection was separately performed with the Varscan software^47^ (minimum read depths supporting variants: 5, minimum read depth:20, p-value threshold for calling variants:0.99), and the VCF files obtained were annotated with ANNOVAR^16^. The pipeline implemented in Galaxy is reported in Supplementary File S1.

Similarly, in the Nanopolish pipeline, reads mapping was executed with BWA-MEM, and the “nanopolish variants” subprogram was used to simultaneously call SNV and indels, which were annotated as described above (-min-candidate-frequency 0.1, -min-candidate-depth = 20).

The ChIPpeakAnno^48^ and BSgenome.Hsapiens.UCSC.hg19 R packages were used to retrieve the sequences flanking the variants detected, in order to evaluate the presence of homopolymer contexts and their potential association with MinION sequencing errors.

### Validation of sequencing variants

The filtered variants were then compared with the results obtained using SS; electropherograms were then analyzed by visual inspection, glass free software for SS analysis data (http://shiny.bat.infspire.org/igcllglass/) and GeneScreen^49^.

For one SNV present at a level below the sensitivity threshold of SS (about 11%), an Allele Specific Oligonucleotide PCR (ASO-PCR) assay was accomplished. The Web-based Allele Specific Primer (WASP) designing tool (http://bioinfo.biotec.or.th/WASP) was used to design the allele-specific primers for detecting the mutated and wild-type site, using the default parameters.

ASO-PCR was performed with Platinum^TM^ Taq DNA Polymerase (Invitrogen), using 100 ng of genomic DNA, in a final volume of 50 uL. Thermal cycling conditions were 95 °C for 30 seconds, 95 °C for 30 seconds, 65 °C for 30 seconds, 72 °C for 30 seconds (35 cycles) and 4 °C hold.

### Data availability

The sequence data from this study have been submitted to the NCBI Short Read Archive (http://www.ncbi.nlm.nih.gov/Traces/sra/sra.cgi) under accession n° SRP133465.

## Electronic supplementary material

Supplementary data

Supplementary Table S2

Supplementary Table S3

Supplementary Table S4

Supplementary information
