# Supplementary material for: Design and MinION testing of a nanopore targeted gene sequencing panel for chronic lymphocytic leukemia
Source: Sci Rep. 2018 Aug 7;8:11798. doi: 10.1038/s41598-018-30330-y (PMC6081477; doi:10.1038/s41598-018-30330-y)
Supplement: Supplementary file 1 — Supplementary data [file 41598_2018_30330_MOESM1_ESM.pdf]

Design and MinION testing of a nanopore targeted gene sequencing panel for chronic lymphocytic leukemia

Paola Orsini[1], Crescenzio F. Minervini[1], Cosimo Cumbo[1], Luisa Anelli[1], Antonella Zagaria[1], Angela Minervini[1], Nicoletta Coccaro[1], Giuseppina Tota[1], Paola Casieri[1], Luciana Impera[1], Elisa Parciante[1], Claudia Brunetti[1,] Annamaria Giordano[1], Giorgina Specchia[1], Francesco Albano[1*]

Supplementary Table S1.

Number of 2D passed reads for each CLL sample, demultiplexed by Metrichor software.

CLL sample	Read Count
**case#1**	3262
**case#2**	5220
**case#3**	2435
**case#4**	3429
**case#5**	4187
**case#6**	4896
**case#7**	1553
**case#8**	5847
**case#9**	5437
**case#10**	3892
**case#11**	3577
**case#12**	2716
**unclassified**	2148

Supplementary Table S2

Error rate analysis of MinION sequencing data for known CLL hotspot mutations.

Supplementary Table S3.

List and annotation of the 256 genomic recurrently mutated positions determined in the CLL patients analyzed; as observed, most of them (91%) were sites of indels, in agreement with the results of error rate analysis. The flanking sequences of these critical genomic positions are reported.

Supplementary Table S4

List and annotation of the 18 non-recurrent variants and known hotposts identified in the CLL patients analyzed.

Supplementary Table S5.

Primers sequences of CLL panel.

Gene target	Forward primer	Reverse primer
**SF3B1 ex14-16**	TCTGTTTTAATGTAGTTTGCTTCTACACCA	CTCATGACTGTCCTTTCTTTGTTTACATTT
**TP53 ex10-11**	ACACCTATTGCAAGCAAGGGTT	AAAAGTCAGCTGTATAGGTACTTGAAGTG
**NOTCH1 ex34**	CTGTGTGTCCATCTCCCTACAA	GTTTCAGAAGATGTATCAAAGCCTTAACAT
**TP53 ex2-9**	TTTTGAAAGCTGGTCTGGTCCTT	TCTCATGCTGGATCCCCACT
**BIRC3 ex6**	CCTGCCATTCTGTTTCCTTC	TGAGCAACTAGCCTGGGATT
**BIRC3 ex7-9**	TGGAAGGAAGTTTGTGAGCA	AGTGCTACCTCTTTTTCGTTCA
**MYD88 ex3-5**	GAGATAATAGTCCTACCTCTGGATTGCT	GCAAATATCGGCTTTTCTCAGATATCTTTG

Supplementary Fig. S1

Visualization of pool 1 (a) and pool 2 (b) purified amplicons by SYBR Safe on agarose gel 1.0%. As shown, digestion with BglII restriction enzyme allowed to verify the successful amplification of all the targets of pool 1 (a), whereas the pool 2 purified amplicons were directly visualized (b). The original images of the two gels are provided as Supplementary Figures S5-S6.

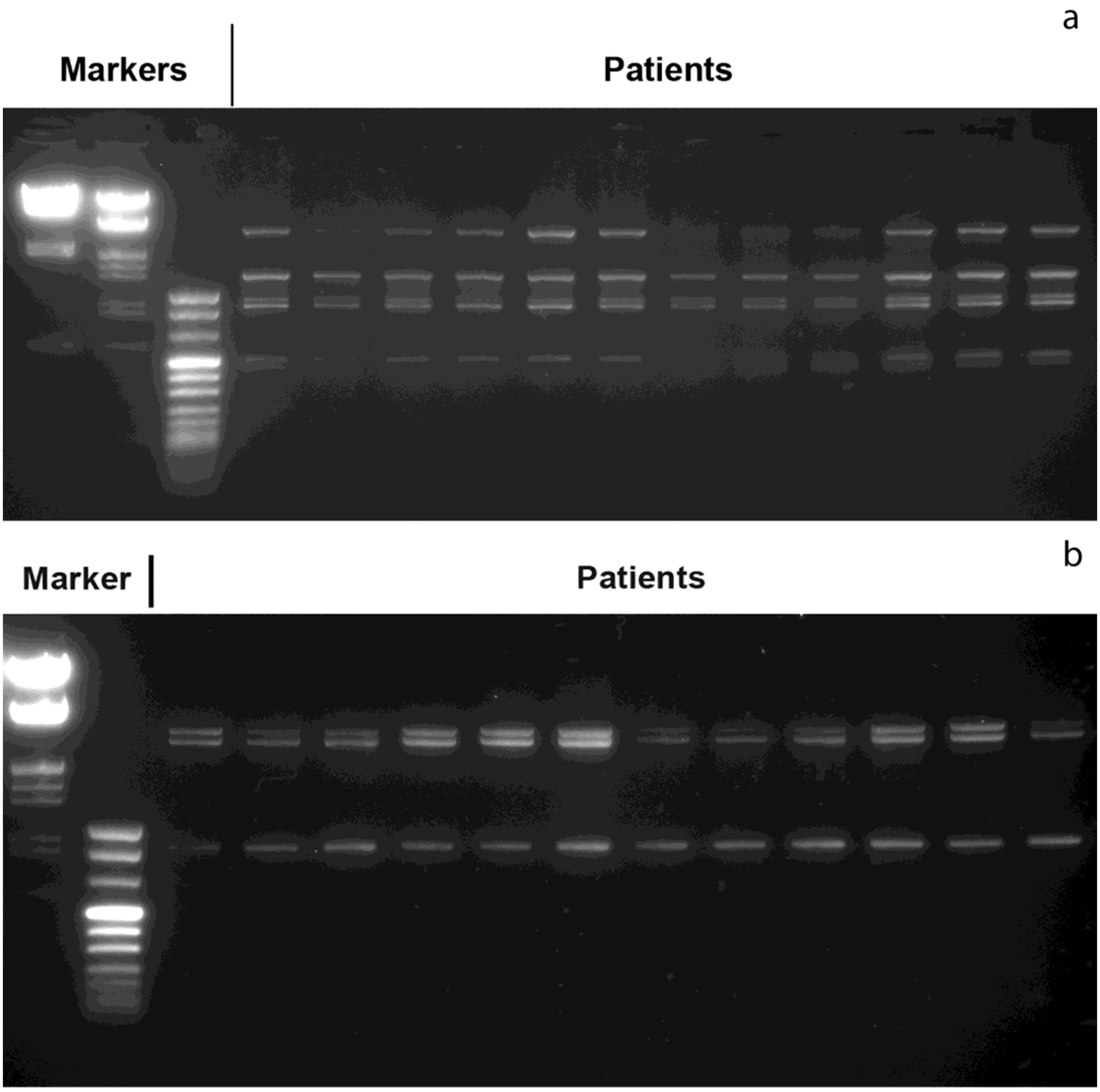

Supplementary Fig. S2

2D Sequence Length *vs* Quality Score. Data from Sequence Length distribution (y-axis) and Mean Quality Score distribution (x-axis) are plotted. 2D reads with the higher quality score are distributed around the mean amplicons size, as expected.

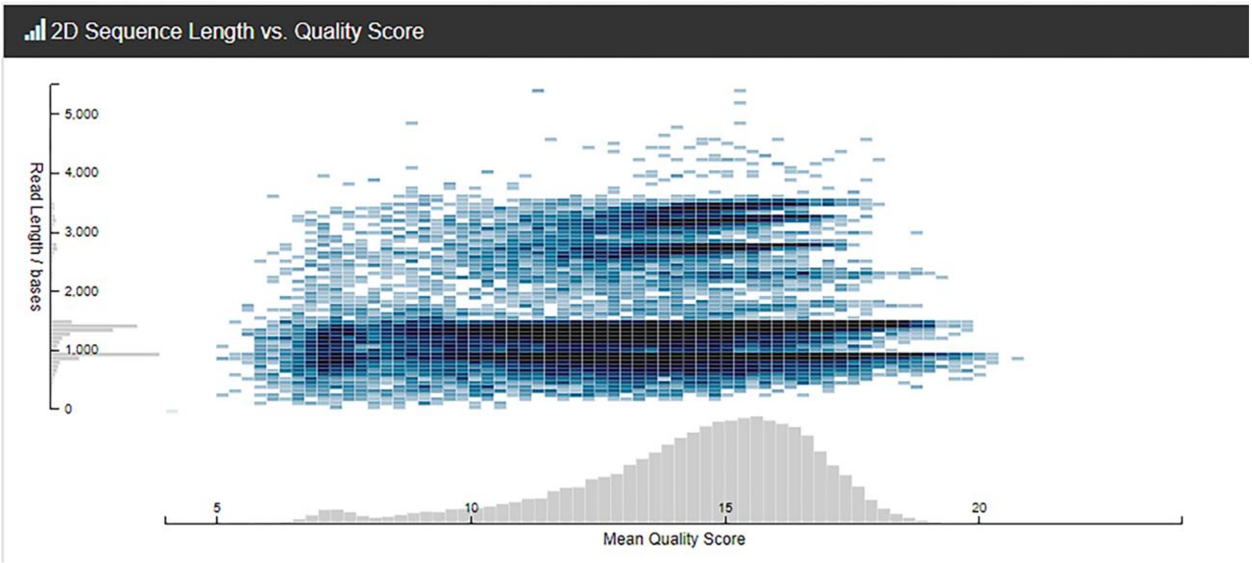

Supplementary Fig. S3

Two mutated cases that were well visible in MinION and were simultaneously confirmed by Sanger Sequencing (SS) (a) or molecular assay (b), in case#9 and case#12, respectively (M: Molecular marker; NC: Negative Control; PC: Positive Control). The original scan of the gel image is provided as Supplementary Figure S7.

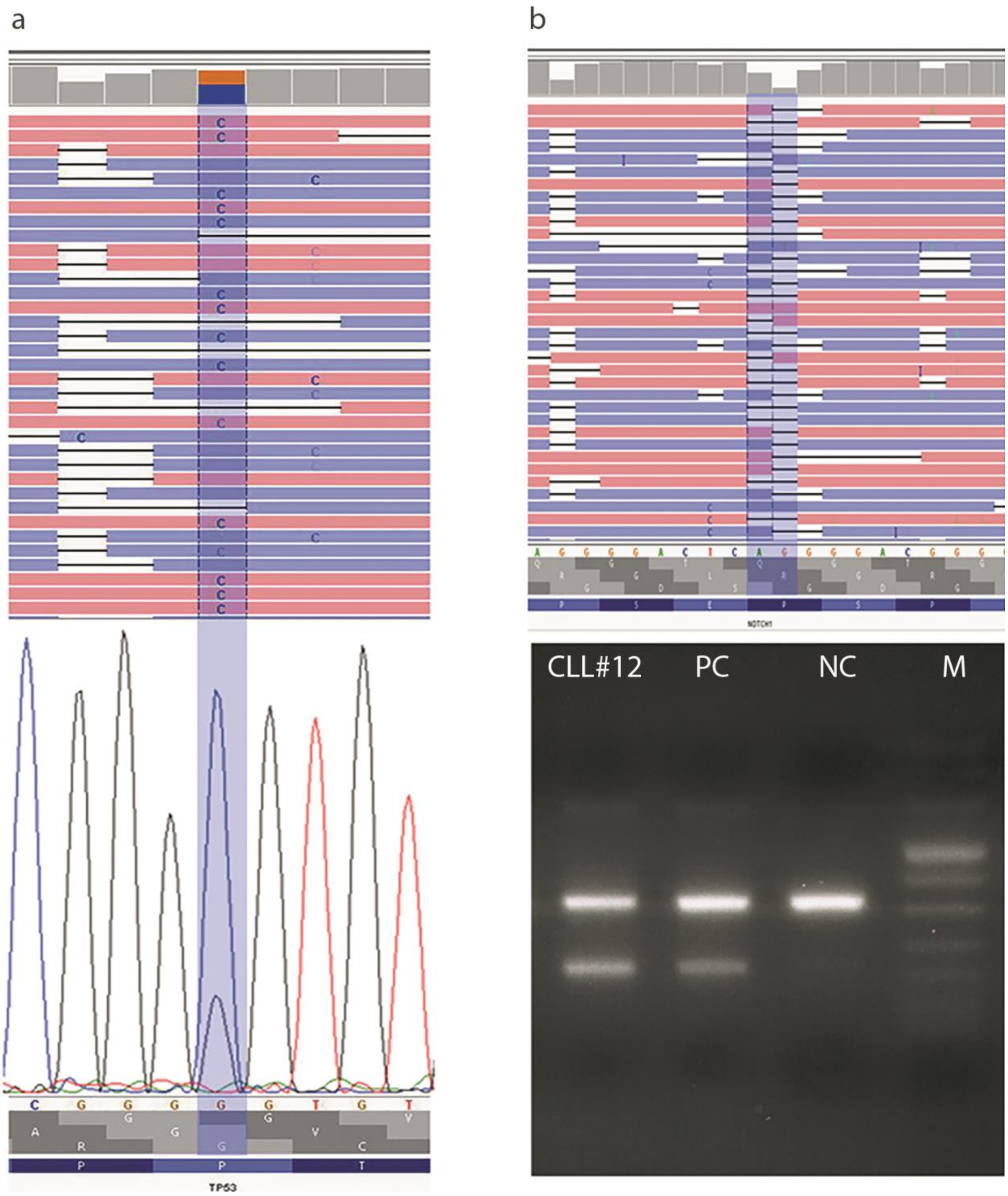

Supplementary File S1

Description of the pipeline implemented in Galaxy.

Pipeline GALAXY

```
Step 1: Map with BWA-MEM
Use a built-in genome        index
Using reference genome:      hg19
Single or Paired-end reads:  Single
Set read groups information? Do not set
Select analysis mode              3.Nanopore 2D-reads mode (-x
ont2d)
Job Resource Parameters      Use default job resource parameters

Step 2: BamLeftAlign
Using reference genome       hg19
Maximum number of iterations 5

Step 3: Generate pileup
Using reference genome       hg19
Where to cap mapping quality 500
Call consensus according to MAQ model? No

Step 4: Varscan
Pileup dataset
Analysis type                single nucleotide variation
Minimum read depth           20
Minimum supporting reads     5
Minimum base quality at a position to count a read    8
Minimum variant allele frequency threshold  0.05
Minimum frequency to call homozygote   0.75
p-value threshold for calling variants 0.99
Ignore variants with >90% support on one strand  yes

Step 5: Varscan
Pileup dataset
Analysis type  insertions and deletions
Minimum read depth  20
Minimum supporting reads 5
Minimum base quality at a position to count a read    8
Minimum variant allele frequency threshold  0.05
Minimum frequency to call homozygote   0.75
p-value threshold for calling variants 0.99
Ignore variants with >90% support on one strand  yes
sample_names   Empty.

Step 7: ANNOVAR Annotate VCF
Variants
Output dataset 'output' from step 4
Gene Annotations   refGene
Annotation Regions  genomicSuperDups phastConsElements46way
```

Annotation Databases 1000g avsift dbsnp_NonFlagged esp6500si_all dbsnp cosmic
Output data type Tabular

Step 8: ANNOVAR Annotate VCF
Variants
Output dataset 'output' from step 5
Gene Annotations refGene
Annotation Regions genomicSuperDups phastConsElements46way
Annotation Databases 1000g avsift dbsnp_NonFlagged esp6500si_all dbsnp cosmic
Output data type Tabular
