# Supplementary material for: Design and MinION testing of a nanopore targeted gene sequencing panel for chronic lymphocytic leukemia
Source: Sci Rep. 2018 Aug 7;8:11798. doi: 10.1038/s41598-018-30330-y (PMC6081477; doi:10.1038/s41598-018-30330-y)

Design and MinION testing of a nanopore targeted gene sequencing panel for chronic lymphocytic leukemia

Paola Orsini[1], Crescenzio F. Minervini[1], Cosimo Cumbo[1], Luisa Anelli[1], Antonella Zagaria[1], Angela Minervini[1], Nicoletta Coccaro[1], Giuseppina Tota[1], Paola Casieri[1], Luciana Impera[1], Elisa Parciante[1], Claudia Brunetti[1,] Annamaria Giordano[1], Giorgina Specchia[1], Francesco Albano[1*]

Supplementary Fig. S4

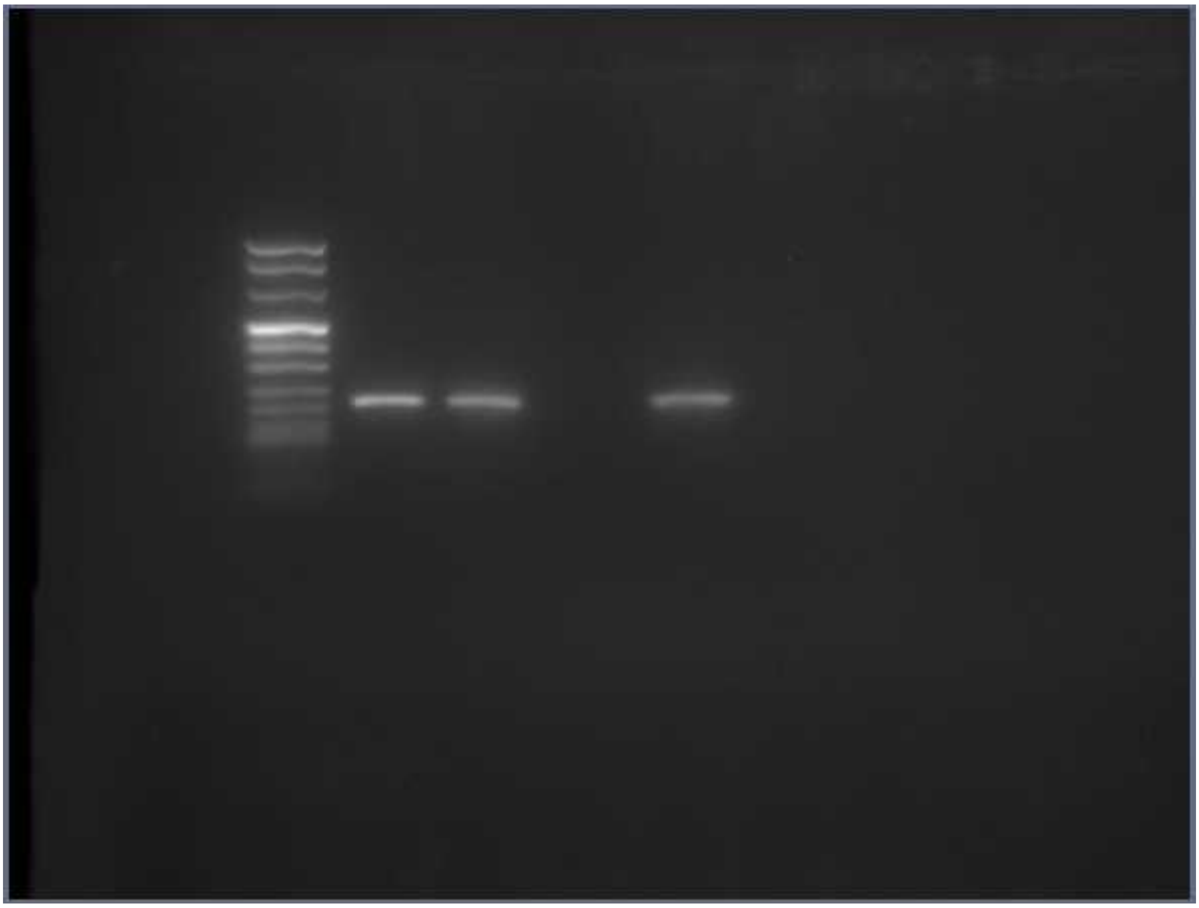

Supplementary Fig. S5

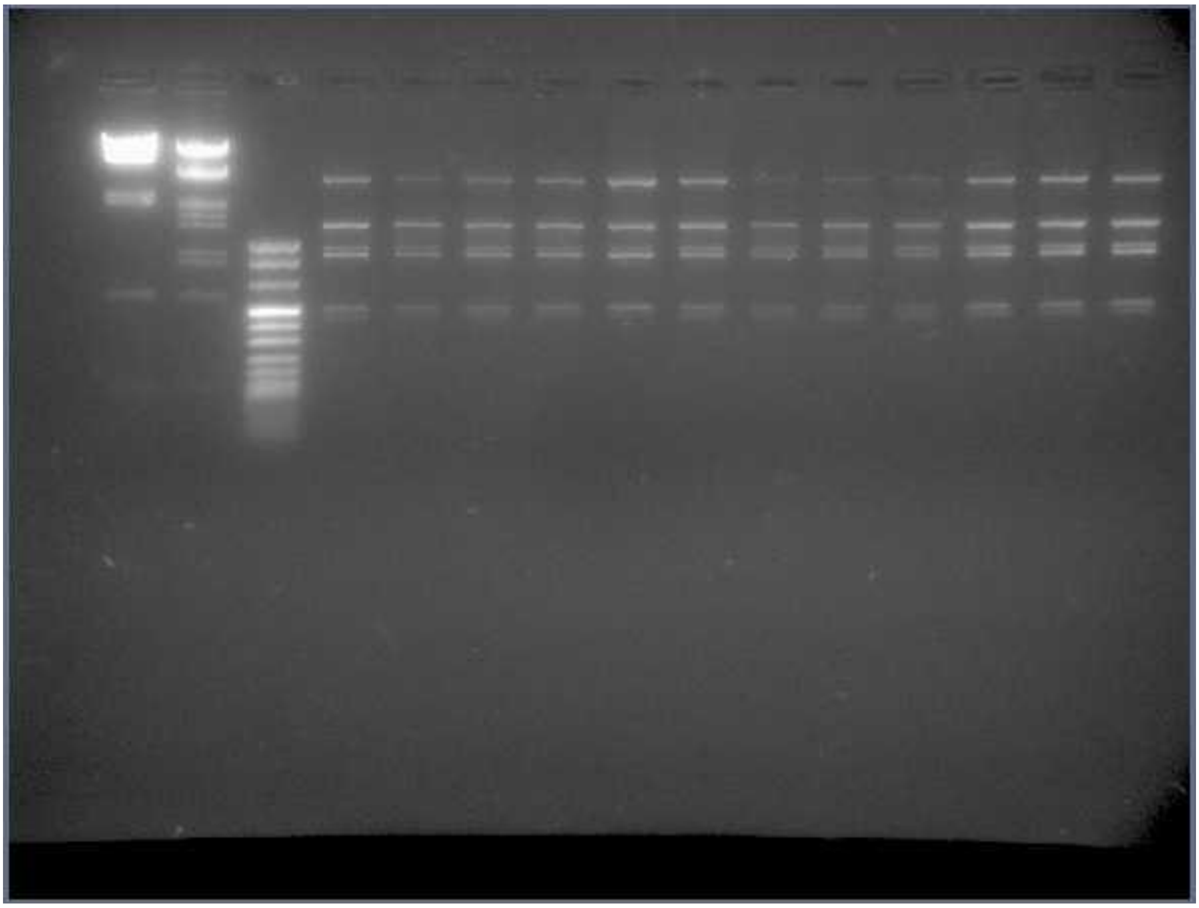

Supplementary Fig. S6

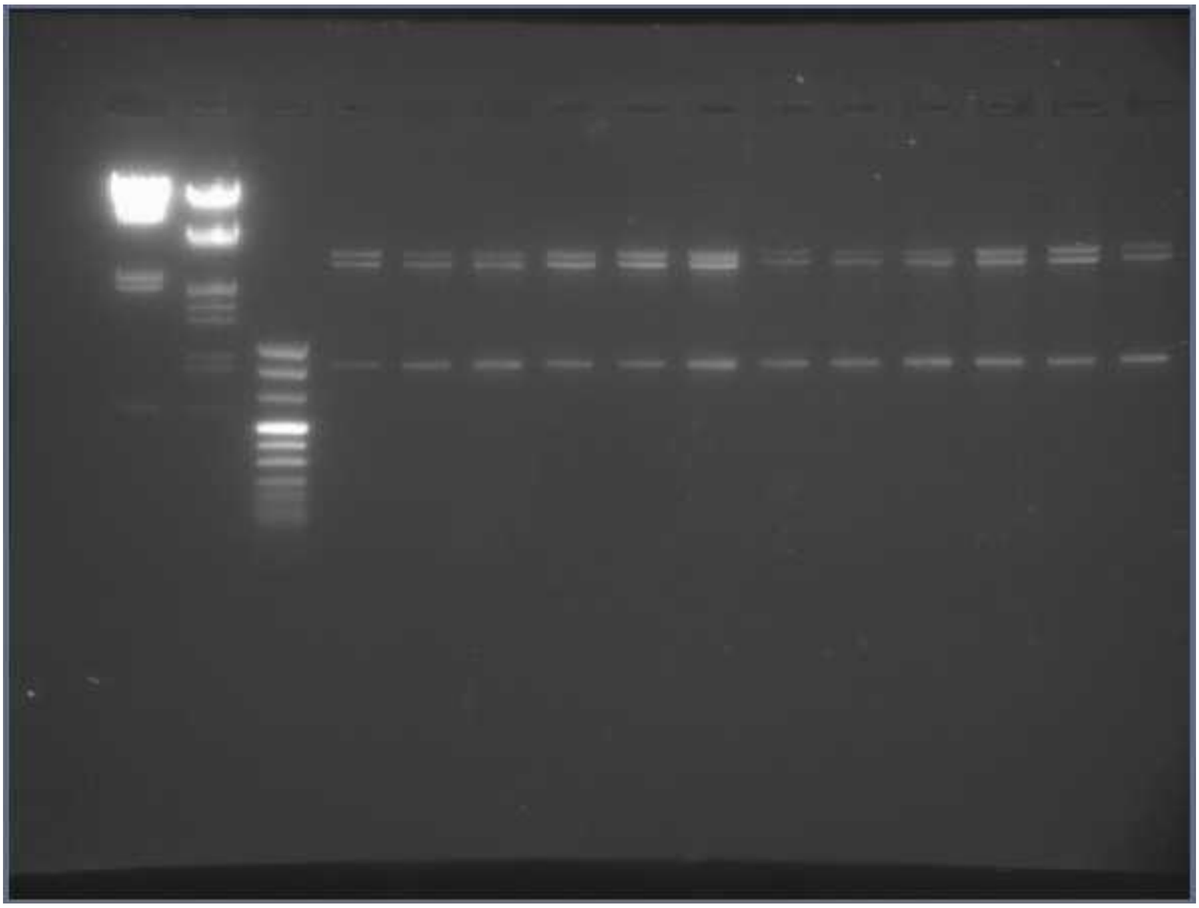

Supplementary Fig. S7

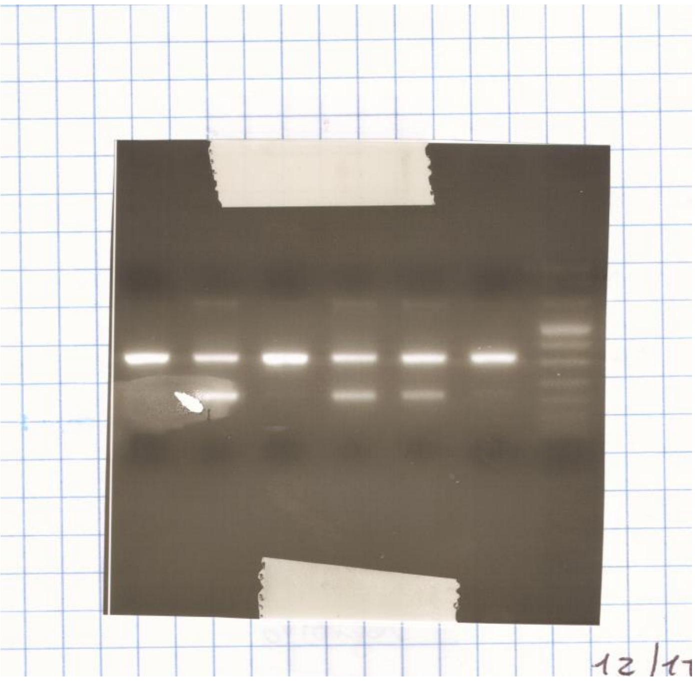

Supplement: Supplementary file 5 — Supplementary information [file 41598_2018_30330_MOESM5_ESM.pdf]
